# O01 Genomic evolution of MRSA ST149 associated with a healthcare worker leading to two outbreaks in a neonatal ICU

**DOI:** 10.1093/jacamr/dlaf118.001

**Published:** 2025-07-14

**Authors:** M A I Asanthi, J Orendi, K Moganeraj, R Yan, M Ganner, M Mirfenderesky, T Lamagni, R Pahalage, R Kerry, A Thomas, S Haidee, O Mutasa, T O’Leary, E Philips

**Affiliations:** University Hospital North Midlands, UK; University Hospital North Midlands, UK; AMRHAI, UK Health Security Agency, UK; AMRHAI, UK Health Security Agency, UK; AMRHAI, UK Health Security Agency, UK; AMRHAI, UK Health Security Agency, UK; AMRHAI, UK Health Security Agency, UK; University Hospital North Midlands, UK; University Hospital North Midlands, UK; University Hospital North Midlands, UK; University Hospital North Midlands, UK; University Hospital North Midlands, UK; University Hospital North Midlands, UK; University Hospital North Midlands, UK

## Abstract

**Background:**

A neonatal ICU (NICU) at a tertiary care NHS trust noticed an increased incidence of MRSA in the period June to December 2024. Screening for MRSA was performed on admission and weekly. Eight babies were found positive for MRSA in screening, sputum or bronchial lavage. All were found to be community-associated (CA) MRSA, with the mother identified as the source in two cases. Two other cases in September and October 2024 were found to be related by WGS: ST 149 CC5, PVL toxin negative, with a less than 5 SNP difference, suggesting a transmission event on the NICU. Of interest, the NICU had identified an outbreak with MRSA ST 149 CC5 in December 2022 to January 2023 when two neonates were found to be positive. Staff screening had revealed a healthcare worker (HCW), a staff nurse, positive for the same MRSA type, who was a new recruit from the Middle East. The cluster had a less than 5 SNP difference: ST 149 CC5-2914.3348.3694. X. The HCW had received topical MRSA decolonization treatment twice and was found to be negative in 5 screenings since 2023, the last in July 2024.

**Investigation:**

Isolation of the same MRSA type (ST149 CC5) in two neonates raised the suspicion that the source of the new transmission event in 2024 might be related to the same HCW again. Comparison by WGS of the two new neonatal isolates in 2024 with the previous isolates from 2022/2023 was performed. This was initially reported as no evidence of a strong genetic relationship between the 2 sets of isolates. Nevertheless, repeat screening of the HCW (nose, throat and perineum) was performed in January 2025, with the nasal swab found positive for MRSA ST149 again. WGS of 20 colony picks indicated that all MRSA ST149 from 2022–25 were related, suggesting that the HCW was the source of two consecutive outbreaks. Following decolonization including decontamination of the home, the HCW achieved three negative MRSA screens and has returned to clinical duties. To ensure long term eradication a decision has been made for follow-up screens until 2 years after the last positive result: monthly for 6 months and quarterly thereafter.

**Conclusions:**

Admission and weekly screening for MRSA in patients admitted to NICU including typing of MRSA is recommended, since the prevalence of CA-MRSA in community and HCWs has increased.

